# Portal Setup: the Key Point in the Learning Curve for Hip Arthroscopy Technique

**DOI:** 10.1111/os.13035

**Published:** 2021-10-18

**Authors:** Li Haipeng, Li Ji, Zhu Juanli, Shi Lijun, Liu Yujie, Li Zhongli, Wang Zhigang, Kong Lu, Li Chunbao

**Affiliations:** ^1^ Department of Orthopedics The Fourth Medical Center, Chinese PLA General Hospital Beijing China; ^2^ Department of Health Services Chinese PLA General Hospital Beijing China

**Keywords:** Hip, Arthroscopy, Learning Curve, Portal Setup

## Abstract

**Objective:**

To analyze the learning curve experience of hip arthroscopy based on patient demographics, surgical time, portal setup time, and postoperative complications and to find the key point in the learning curve.

**Methods:**

From May 2016 to February 2019, a prospective study on the learning curve experience of hip arthroscopy was performed in our hospital. We evaluated the first 50 consecutive hip arthroscopy procedures performed by a single surgeon. There were nine females and 41 males with a mean age of 30.8 years. We divide the patients into early group and late group according to the date of their operation, with each group including 25 patients. Data on patient demographics, types of procedure, surgical time, portal setup time, and postoperative complications were collected. Functional scores were assessed with the modified Harris Hip Score (mHHS).

**Results:**

Patients were followed up for 16.4 months on average (range, 13–27 months). The early group of patients had a mean age of 35.2 years and the late group a mean age of 26.5 years. The most common procedures performed for the early group were debridement (17 patients, 68%), and in the late group, most patients underwent labral repair (18 patients, 72%). Mean total surgical time was 168 min for the early group and 143 min for the late group, and there was no statistically significant difference between two groups. The portal setup time in the early group and late group was 40.2 ± 12.4 min and 18.5 ± 6.2 min, respectively (*P* < 0.001), and the portal setup time was significantly longer in the early group. Further analysis of the learning curve of portal setup showed that the average portal setup time was not statistically significant changed after 30 cases. There were six complications including iatrogenic cartilage injury and iatrogenic labrum injury in the early group and five complications including perineal crush injury and nerve stretch injury in the late group. The functional score of patients in the late group was significantly higher than that in the early group during follow‐up.

**Conclusion:**

The steep learning curve of hip arthroscopy is mainly caused by the challenge of portal setup and portalrelated complications were more common in the early group than in the late group. Surgical time is not an effective indicator for evaluating progress on the learning curve of hip arthroscopy.

## Graphical abstract

This study analyzed the learning curve experience of hip arthroscopy based on the data of 50 patients, and found that the steep learning curve was mainly caused by the challenging of portal setup and portal‐related complications were more common in the early stage.

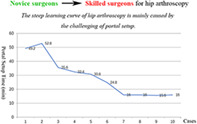

## Introduction

Hip arthroscopy is a rapidly developing method for treating various hip diseases over the past decade, and increasing numbers of surgeons are now performing hip arthroscopy as a routine procedure^1^. Hip arthroscopy is now used for treating several pathologies, such as femoroacetabular impingement (FAI), acetabular labral tear, snapping hip, septic arthritis of the hip, and loose body^2^, ^3^. However, hip arthroscopy is a demanding procedure with a steep and long learning curve in historical review of arthroscopic surgery^4^. It was first performed by Burman in 1931, but the development of hip arthroscopy has been relatively delayed compared to knee arthroscopy or shoulder arthroscopy. Burman stated that “it is manifestly impossible to insert a needle between the head of the femur and the acetabulum.”^5^

Access to the hip joint is substantially more challenging than it is for the knee or shoulder joint. This is due to the deep engagement of the femoral head with the acetabulum, accompanied by a thick fibrous capsule and muscle capsule that prevent the joint from opening sufficiently to make the use of arthroscopic instruments easier in the operation^2^. Although custom length arthroscopy, intra‐articular razor, and cauterization equipment have comfortably extended the reach of the surgeon to the hip joint, the technical complexity of this process requires advanced skills that differ from those required for arthroscopic knee or shoulder surgery. Therefore, hip arthroscopy is widely recognized as a technically demanding procedure and is almost universally described as having a difficult or “steep” learning curve. The learning curve of hip arthroscopy is much steeper than knee arthroscopy^6^ and shoulder arthroscopy^7^. There is the need to understand the various factors that influence the acquisition of skills and perform a safe and effective hip arthroscopy^8^. The study about the learning curve of hip arthroscopy is helpful to highlight basic pitfalls in the training of novice surgeons for hip arthroscopy.

There have been several reports of hip arthroscopy involving the learning curve of hip arthroscopy. In most studies, surgical time, clinical outcomes, reoperation, and complication rates are used to evaluate the learning curve^9^, ^10^, ^11^. Konan^12^ prospectively reviewed the first 100 hip arthroscopic procedures and found that there was a 46% decrease in operative time from the first 10 cases to the remaining 90 operations, representing a gradual learning process. Lee^9^ performed a retrospective study of 40 consecutive patients who underwent hip arthroscopy. They evaluated the learning curve based on patient‐oriented outcomes, and found that experience of approximately 20 cases is required to achieve satisfactory outcomes in terms of clinical outcomes. Kautzner^1^ evaluated 150 hip arthroscopy procedures performed by a single surgeos and found a statistically significant decrease of complication rate with more procedures performed.

In our clinical practice, we noticed that the portal setup was more difficult in arthroscopy surgery. Therefore, we designed a prospective study to evaluate the first 50 hip arthroscopy procedures performed by a surgeon in our department. Types of procedure, surgical time, portal setup time, incidence of complications, and a subjective functional score were evaluated. Based on this analysis, we estimated the learning curve of hip arthroscopy to find the key point in the learning curve. The objective of the current study was to: (i) analyze the changes of surgical time, portal setup time during the learning curve; (ii) evaluate the experience of the learning curve on the types of complication; and (iii) provide the impact of the learning curve on the functional score of patients.

## Patients and Methods

### Subjects

The first 50 hip arthroscopic procedures performed by the senior author (HP Li) from May 2016 to February 2019 were prospectively evaluated. Approval was obtained from our ethics committee. The inclusion criteria for hip arthroscopy were as follows: (i) patients presenting hip pain who consulted with a senior arthroscopic doctor during the admission and then underwent strict physical examination; (ii) hip pain was not relieved by conservative treatment for at least 3 months; (iii) magnetic resonance imaing (MRI) or X‐ray findings of intraarticular pathology of hip (FAI, acetabular labral tear, or loose body). The exclusion criteria were as follows: (i) signs of severe osteoarthritis on a preoperative X‐ray; (ii) hip joint line narrowing to under 4 mm; (iii) infection in hip joint; and (iv) tumor in hip joint.

### Groups

To examine the different stages of the learning curve, the initial 50 consecutive hip arthroscopic procedures performed by the senior author were chronologically stratified into two groups (the early group: cases 1–25; and the late group: cases 26–50) according to the date of procedure^9^. The early group and late group were of equal size with 25 patients in each group.

The senior author was experience in knee and shoulder arthroscopy, having performed more than 200 arthroscopic knee and shoulder procedures before starting to perform hip arthroscopies, was familiar with hip‐preserving surgery using standard approaches for surgical hip dislocation and with all hip arthroscopy‐related instruments. The senior author also completed cadaveric hip arthroscopy courses, and was trained in X‐ray and MRI evaluation and proper clinical examination for a diagnosis of symptomatic hip pain.

### Surgery Technique

#### Anesthesia and Position

After general anesthesia, patients were placed in a supine position with their two lower limbs placed on the traction bed and immobilized, and the perineum protected. The skin of the affected region was disinfected conventionally, and a sterile drape laid. Traction was applied to the lower limb on the affected side, with abduction and intorsion of the hip joint.

#### Portal Setup

Under the C‐arm X‐ray, when the joint space was retracted apart by 8–10 mm, the conventional anterolateral portal was established. The arthroscope was then inserted, and the medioanterior portal established. The articular capsule was opened and the anterolateral and medioanterior portals communicated with each other. The arthroscope was placed into the central compartment of the hip joint.

#### Repair Intra‐articular Pathology

The acetabular labrum, cartilage of the acetabulum and femoral head, the acetabular fossa, and the round ligament were examined. Resection, trimming, or suturing with a suture anchor was performed based on the tissue of the damaged acetabular labrum. Next, the retractors on the lower limb were relaxed, and the affected acetabulum flexed by 35–45°. The hip arthroscope was placed into the peripheral compartment. The non‐weight‐bearing surface, femoral head–neck junction, and hip joint capsule were examined. Depending on the morphology of the cam, a longitudinal incision was made on the articular capsule along the long axis of the femoral neck. That is, a T‐shaped incision was made in the articular capsule to fully expose the bony growth. The hyperplasia was removed using a drill, and the impingement was observed dynamically under the arthroscope to determine the abrasion degree of the osteophyte.

#### Close Incisions and Record Information

In the last step, a figure‐of‐8 suture was performed to close the incisions in the articular capsule and skin. The surgical time, traction time, and portal setup time were recorded and evaluated. During surgery, the type of reconstructive procedure performed was also recorded.

### Postoperative Rehabilitation

At 1‐day post‐surgery, patients were instructed on partial weight‐bearing walking using two crutches as long as it was tolerable. At 1–4 weeks post‐surgery, passive movement of the hip joint continued along with active movement of the hip joint within the acceptable range. The normal mobility of the joint was gradually restored (anteflexion, backward extension, abduction, adduction, eversion, and pronation). Full weight‐bearing walking was also gradually restored within the acceptable range. Patients began full weight‐bearing walking at 4‐weeks post‐surgery. The patients were able to walk normally 3 months after surgery and began such exercises as jogging and stair climbing.

### Follow‐up

#### Perioperative Information

The surgical time and portal setup time were recorded and evaluated. The portal setup time included the establishement of the anterolateral portal, medioanterior portal, and the arthroscopic capsulotomy until the anterolateral and medioanterior portals communicated with each other.

During the surgery, the types of procedure performed were also recorded. The overall complication rate was collocated and the types of complication were analyzed after surgery.

#### Modified Harris Hip Score (mHHS)

Patients were followed up in the outpatient department at 6, 12, and 24 weeks after surgery, and every 6 months thereafter. Functional scores were assessed with the modified Harris Hip Score (mHHS). Patients' subjective satisfaction with surgery was assessed based on a scale of 0 to 100, with higher scores indicating better function: scores >90 points were rated as excellent results, 80–90 points as good results, 70–80 points as fair results, and <70 points was deemed a clinical failure.

#### Statistical Analyses

An independent observer not involved in the surgery performed all patient evaluations. The observer was a doctor working in our department who had an in‐depth knowledge of the disease, the surgery, and the scoring systems.

All statistical analysis was performed using SPSS for Windows version 16.0 (SPSS, Chicago, IL). Data were expressed as mean ± standard deviation (SD). A paired *t*‐test was used to compare the pre‐ and postoperative scores. Independent‐sample *t*‐tests were used to compare the quantitative data between the two groups, while qualitative data of the two groups were compared using the chi‐squared test. Statistical significance was set at *P* < 0.05.

## Results

### General Results

From May 2016 to February 2019, the first 50 hip arthroscopic procedures were performed by the senior author (HP Li). There were 41 males and nine females, with the average age of the patients being 30.8 ± 12.7 years (range, 15–67 years), and the early group had a mean age of 35.2 ± 15.5 years and the late group a mean age of 26.5 ± 7.1 years. Complete follow‐up was achieved for all patients in both groups. Patients were followed up for 16.4 months on average (range, 13–27 months).

For the types of procedure, in the early group, 17 patients underwent isolated hip arthroscopic debridement (68%), five cases included labral repair and acetabuloplasty (20%), and six patients had Cam plasty (24%). In the late group, 18 patients underwent labral repair and acetabuloplasty (72%), 15 patients had Cam plasty (60%), and six patients had isolated hip arthroscopic debridement (24%).

#### Surgical Time and Portal Setup Time

The average surgical time in the early and late groups was 168.0 ± 56.8 min and 154.4 ± 54.4 min, respectively; this difference was not statistically significant (*P* = 0.3918). Surgical time was divided into portal setup time and surgical operation time. The portal setup time in the early group (40.2 ± 12.4 min) was significantly longer than that in the late group (18.5 ± 6.2 min; *P* < 0.001), and the portal setup time in late group was 46% of the early group. In contrast, the operation time between the two groups did not differ significantly (early group: 127.8 ± 53.5 min; late group: 135.9 ± 51.6 min; *P* = 0.5894).

#### Subgroups Analyses for Portal Setup Time

**Fig 1:**
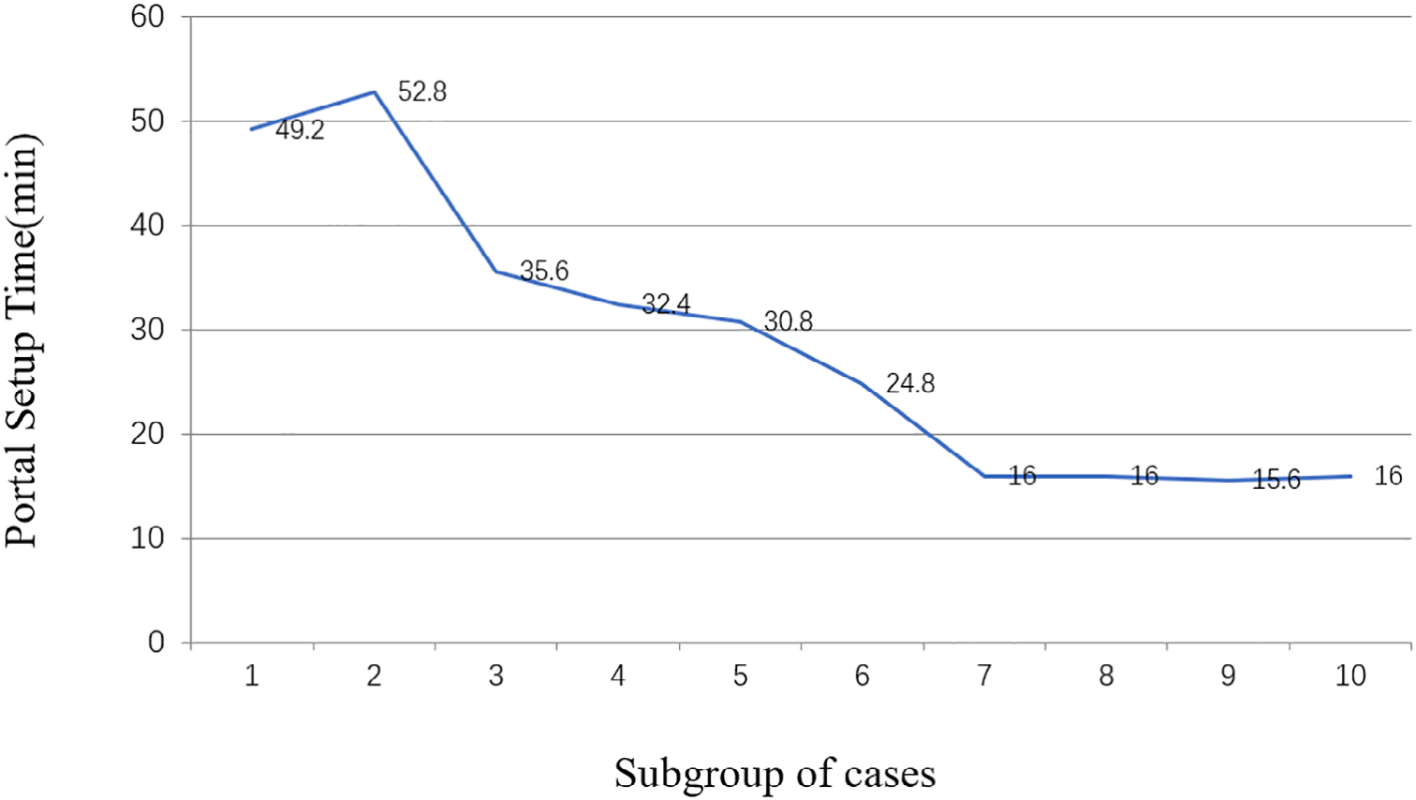
Learning curve for portal setup. The initial 50 patients were divided into 10 subgroups with equal size. The average portal setup time of each subgroup were analyzed, and the result showed that the average portal setup time was consistent after subgroup seven. Therefore, the learning curve of portal setup was established.

To further analyze the learning curve of portal setup, 50 patients were divided into 10 subgroups according to the sequence of operation time. The average portal setup time of each subgroup was 49.2 ± 12.3 min, 52.8 ± 12.0 min, 35.6 ± 7.0 min, 32.4 ± 5.6 min, 30.8 ± 5.8 min, 24.8 ± 9.1 min, 16 ± 1.5 min, 16 ± 5.7 min, 15.6 ± 3.3 min, 16 ± 4.4 min, respectively (Fig. 1). Analyses of the subgroups showed that the average portal setup time was not statistically significant and changed after 30 cases; therefore, the learning curve of portal setup was established.

#### Modified Harris Hip Score (mHHS)

The preoperative mHHS score was 42.2 ± 6.7 and 41.6 ± 5.5 in the early and late groups, respectively (*P* = 0.7486). The postoperative scores of the two groups at the last follow‐up were 76.8 ± 7.8 and 84.1 ± 6.5, respectively (*P* = 0.0008). Compared to the preoperative score, the mHHS score was significantly improved in both the early group (*t* = −68.14, *P* < 0.001) and in the late group (*t* = −54.23, *P* < 0.001) at the last follow‐up. The functional score of patients in the late group was significantly higher than that in the early group during follow‐up.

The patients' subjective satisfaction score was 77.7 ± 7.1 and 84.9 ± 6.5 in the early and late groups, respectively (*P* = 0.0005). In the early group, 36% of patients had scores that were excellent or good, while in the late group, 76% of patients fell into the excellent or good category.

#### Complication

The incidence of complications was similar in the early and late groups, with six cases and five cases, respectively, but the types of complications differed (Table 1). In the early group, complications were mainly iatrogenic cartilage injury and iatrogenic labrum injury. In the late group, traction‐related complications predominated, including perineal crush injury and nerve stretch injury.

**TABLE 1:** Types of complications of the early and late groups

	Early group (Cases)	Late group (Cases)
Iatrogenic cartilage injury	3	0
Iatrogenic labrum injury	1	1
Nerve stretch injury	1	2
Perineal crush injury	0	2
Heterotopic ossification	1	0

## Discussion

With increasing worldwide use, hip arthroscopy is a surgical method with satisfactory clinical results, and developing an understanding of its learning curve is important. In this prospective study, the first 50 hip arthroscopy surgery patients in the first author's practice were analyzed to measure indicators, such as types of procedure, surgical time, portal setup time, incidence of complications, postoperative function scores, and satisfaction scores of patients, that might elucidate the learning curve of hip arthroscopy.

### Learning Curve of Surgical Time and Portal Setup Time

Surgical time is frequently considered an important factor in evaluating the learning curve of hip arthroscopy^1^, ^9^. Four studies included in a systematic review by Hoppe^4^ chose operation time to measure the hip arthroscopy learning curve and found that the operation time of the late group was significantly reduced compared to the early group. However, surgical time is affected by many factors^1^. In this study, the average surgical time in the early and late groups was 168.0 ± 56.8 min and 154.4 ± 54.4 min, respectively, and this difference was not statistically significant.We analyzed the types of procedure during the learning curve and found that 68% of patients in the early group had isolated hip arthroscopy, while in the late group, 72% of patients had a labrum repair and 60% of patients underwent cam plasty. Clearly, the surgical time required in the late group was correspondingly prolonged given the increased complication of the operations performed compared to the early group. Therefore, surgical time cannot be used as the only indicator to evaluate the learning curve of hip arthroscopy.

We further divided the whole surgical time into portal setup time and operation time. In the early group, the portal setup time was 40.2 ± 12.4 min and in the late group it was 18.5 ± 6.2 min. There was a significantly longer time for portal setup in the early group. Thus, we suggest that the portal setup is critical in the early stage of the learning curve. Hip arthroscopy surgery is more difficult in the setup of the portal than the knee and shoulder surgery due to the anatomical characteristics of the hip joint^12^. In addition, the articular capsule incision technique is an important part of the hip arthroscopy operation. The author did not perform articular capsule incision or only performed an incomplete incision in the early‐stage surgeries, prolonging the time of the instrument entering the joint cavity. Kautzner^1^ similarly did not observe a gradual decrease in operation time during the learning curve in a prospective study and suggested that with the increase of experience in hip arthroscopy surgery, the time required to establish the portal is significantly shortened. We further analyzed the learning curve of the portal setup, and divided 50 patients into 10 subgroups. The result shown that the average time for portal setup was not statistically significant but changed after 30 cases; therefore, the learning curve of portal setup was established. Our findings are consistent with other studies. Hoppe^4^ reported a systematic review on the learning curve for hip arthroscopy, and they found that three of the six articles reported statistical significance, whereas an additional two studies showed trends supporting 30 cases as the number needed for the cutoff point of the learning curve for hip arthroscopy.

### Types of Complication During Learning Curve

The complication was another important indicator for the learning curve of hip arthroscopy. Complications of hip arthroscopy include traction‐related injuries, cartilage injuries, glenoid injury, infections, iatrogenic hip instability, proximal femoral fractures, fluid extravasation, deep vein thrombosis, and pulmonary embolism^13^. In this study, no significant differences occurred in the number of postoperative complications between patients in the early and late groups, but the types of complications differed. Complications in the early group were mainly portal‐related, such as cartilage injury and glenolabial injury, while in the late group, traction‐related injuries, such as perineal crush injury and lower extremity nerve symptoms, predominated.

According to the literature, iatrogenic cartilage or glenolabial injuries most commonly occur during the portal setup. The hip joint is deeper than the shoulder or knee joint, and therefore the surrounding muscle is thick, and the joint capsule is hypertrophic; these factors create higher technical requirements for the portal setup. During the learning curve for hip arthroscopy, the incidence of cartilage injury and glenolabial injury ranges from 0.7% to 20%^13^. These iatrogenic injuries are unavoidable at the early stage of learning, and this was also true in this study. The early cases in this study were relatively simple, and the requirements for traction distance were not high, so traction‐related complications were relatively few. Hip arthroscopy cases during the late stage were relatively complicated, requiring higher traction and longer traction time, which can easily lead to traction‐related complications. The total incidence of traction‐related neurological disorders is reported to be between 0.48% and 20%^14^. However, permanent nerve damage under hip arthroscopy is relatively rare, and most complications are temporary neurological dysfunction or neurological paralysis. Our results are consistent with these reports.

### The Impact of Learning Curve on the Function

Clinical outcomes after hip arthroscopy are major indicators of success. Good functional outcomes are based on learning the basic surgical skills. In this study, we also evaluated functional outcomes after the learning curve to achieve appropriate technical skills. We found that the functional improvement of patients in the late group was better than in the early group, and the patients' subjective satisfaction with surgery was also significantly improved.

In addition to the advancement of surgical technique, this may be related to the selection of surgery indications. Hip arthroscopy was used in clinic as a diagnostic tool in the early days for unexplained hip or groin pain that could not be diagnosed with the traditional imaging examinations. With advances in technology, the indications for hip arthroscopy surgery have gradually been refined and no longer include loose body of the hip joint or repair to the damage of the teres ligament and cartilage of the hip joint^14^. More recently, the number of hip arthroscopy operations worldwide has increased with the introduction of FAI concept. At present, the most common indications for hip arthroscopy are FAI and labrum injury^15^.

In this study, the cases of hip arthroscopy performed by the author followed the characteristics of the development of hip arthroscopy technology. The indications for hip arthroscopy in the early stage were relatively wide and the patients were more variable in age. As the number of surgeries increased, the understanding and diagnosis of hip diseases also improved, resulting in stricter surgical indications. FAI has become the main surgical indication for hip arthroscopy. Many studies have shown that arthroscopic treatment for FAI patients can significantly improve prognosis, especially in terms of hip pain and function^16^.

Several limitations of this study should be addressed. First, this study performed a learning curve of a single surgeon for a relatively small sample size. Therefore, the results could be biased. Second, the diagnosis of the clinical case was inconsistent. The operation time difference for different diseases were not comparable. Therefore, it was difficult to come to an accurate conclusion.

Future studies should provide consistent and comparable cases to evaluate learning curve of hip arthroscopy, thus providing an improved understanding of the learning curve.

### Conclusions

Portal setup was one of the key points for the steep learning curve of hip arthroscopy. The portal setup time was significantly decreasing during the learning curve, and the learning curve of portal setup was established after 30 cases. Portal‐related complications were more common in the early stage of the learning curve. Surgical time is not an effective indicator for evaluating progress on the learning curve of hip arthroscopy. This study is helpful to highlight basic pitfalls in the training of novice surgeons for hip arthroscopy.

## Disclosure

The author(s) declared no potential conflicts of interest with respect to the research, authorship, and/or publication of this article. This study was funded by the Key Basic Research Project of Basic Strengthening Plan (Grant Number 2020‐jcjq‐zs‐264).

## Authors' Contribution

Li Haipeng and Li Ji contributed equally.
